# Transcriptomic Analysis Reveals Molecular Mechanisms of Fleeing, Adhesion, and Thanatosis Patterns in Sea Cucumber *Apostichopus japonicus*

**DOI:** 10.3390/biology15120975

**Published:** 2026-06-22

**Authors:** Guo Wu, Hengye Wu, Xiajing Wang, Qiang Gao, Chong Zhao

**Affiliations:** 1 Key Laboratory of Mariculture & Stock Enhancement in North China’s Sea, Ministry of Agriculture and Rural Affairs, Dalian Ocean University, Dalian 116023, China; 2 College of Marine Science and Environment, Dalian Ocean University, Dalian 116023, China

**Keywords:** sea cucumbers, stress response patterns, fleeing, adhesion, thanatosis, molecular mechanisms

## Abstract

Sea cucumbers (*Apostichopus japonicus*) show fleeing, adhesion, and thanatosis patterns upon exposure to various stressors. However, the molecular mechanisms underlying these contrasting stress response patterns remain largely unknown. In the present study, we performed a transcriptomic analysis of coelomocytes on stressed sea cucumbers to elucidate the potential molecular mechanisms. The RNA-seq results revealed that several matrix metalloproteinase (*MMP*) family genes, along with *HTR4*, *HRH2*, and *ADRA1D* (which are involved in neuroactive ligand–receptor interactions), were significantly upregulated in the fleeing pattern. These genes may facilitate rapid movement. In the adhesion pattern, *PHKA* and *PGK* were significantly downregulated, and the differentially expressed genes (DEGs) were significantly enriched in the longevity regulating pathway, accompanied by downregulation of *KRAS* and *HSPA1*. These genes and the pathway may be involved in the reallocation of energy resources during the adhesion pattern. In the thanatosis pattern, DEGs were significantly enriched in the MAPK signaling pathway (including upregulation of *ANGPT1* and *FGFR1*) and in the Rap1 and Ras signaling pathways (with downregulation of key genes: *RAPGEF4*, *RRAS2*, and *RaLA*). These genes potentially contribute to sustaining the thanatosis pattern. These transcriptomic profiles provide novel insights into the distinct molecular signatures underlying each stress response pattern in *A. japonicus*.

## Simple Summary

As stressors are ubiquitous in the environment, organisms need to regulate their behavioral and physiological responses to cope with such challenges. Sea cucumbers (*Apostichopus japonicus*) are invertebrates lacking a centralized brain, and they exhibit the fleeing, adhesion, and thanatosis patterns in response to different stressors. We performed a transcriptomic analysis of coelomocytes on stressed sea cucumbers to elucidate the potential molecular mechanisms underlying these stress response patterns. This study can deepen our understanding of stress response patterns in sea cucumbers and provide valuable insights into the stress responses of invertebrates that lack a centralized brain.

## 1. Introduction

As stressors are ubiquitous in the environment, organisms need to regulate behavioral and physiological responses to resist stressors [1]. These responses are crucial for their survival [2]. Fleeing, freezing and thanatosis are common stress response patterns, exhibiting distinct behavioral and physiological alterations [3,4,5]. Sea cucumbers are non-centralized brain invertebrates [6]. The natural geographical range of *Apostichopus japonicus* extends across the coasts of China, Japan, Korea, and the Russian Far East [7]. Furthermore, they actively farmed in aquaculture due to their high economic value [8]. Intensive aquaculture mitigates certain environmental stressors, but some stressors are inevitable. The death of some individuals in a sea cucumber population exposes the remaining individuals to conspecific carcasses, which is a stressor that exists in both natural and aquaculture environments. In aquaculture environments, aquafarmers use pressurized water jets to rinse feces from the bottom of the culture tanks. This inevitably subjects sea cucumbers to the seawater rinsing. To grade by size, aqua-farmers place sea cucumbers on a mesh sieve and shake it back and forth so that smaller individuals fall through while larger ones remain. This process inevitably subjects sea cucumbers to mechanical disturbance. Our previous study demonstrated that *A. japonicus* exhibits fleeing, adhesion and thanatosis patterns to different stressors [9]. Escape responses are characterized by faster movement speeds and regulated by the concerted action of multiple neurotransmitters [10,11]. During the fleeing pattern, sea cucumbers exhibit increased movement speed, along with elevated concentrations of serotonin and norepinephrine [9,11]. However, it remains unclear whether additional neurotransmitters contribute to this response and what mechanisms sustain continuous rapid crawling. In the adhesion pattern, increased adhesion capacity is associated with elevated cortisol and reduced Gamma-aminobutyric acid (GABA) levels [9]. Upon exposure to stressors, both cortisol and GABA participate in the adaptive reallocation of energy metabolism [12,13]. Whether the energy allocation changes during this stress response pattern remains unknown. In the thanatosis pattern, sea cucumbers become motionless and contract into a spherical shape, accompanied by a reduction in glucose levels [9,14]. This contractile response requires sustained muscle contraction. The signaling pathways regulating muscle contraction and glucose fluctuations remain unclear. These unanswered questions limit our in-depth understanding of stress response patterns in sea cucumbers.

Molecular processes are closely related to behavioral and physiological responses [15]. The gene expression serves as the basis for linking genotype to phenotype, and RNA synthesis constructs complex gene expression networks [16]. Transcriptome analysis has become a common tool in the stress physiology of marine organisms [17,18,19], because it enables the genome-wide identification of differentially expressed gens and provides crucial information on the molecular determinants of phenotypic responses [20]. Based on these advantages, this approach has been increasingly utilized to investigate the molecular mechanisms of stress response in sea cucumbers exposed to various stressors [21,22,23]. Chang et al. (2022) used transcriptomic sequencing to elucidate how sea cucumbers (*A. japonicus*) respond to heat stress at the molecular level [21]. Similarly, Jiang et al. (2024) applied this method to reveal the changes in energy metabolism in sea cucumbers (*Holothuria moebii*) exposed to hypotonic environment [22]. Therefore, transcriptomics is a suitable method to elucidate the molecular mechanisms of fleeing, adhesion and thanatosis patterns.

The present study aimed to comparatively elucidate the molecular mechanisms underlying fleeing, adhesion and thanatosis patterns using the method of transcriptomic sequencing. Our findings extend current knowledge of how sea cucumbers respond to stressful stimuli at the mechanistic level.

## 2. Materials and Methods

### 2.1. Experimental Animals and Housing Conditions

The sea cucumber individuals (~10 g) used in this experiment were transported from Dalian Xinyulong Marine Organisms Seed Industry Technology Co., Ltd. (Dalian, China) to the Key Laboratory of Mariculture & Stock Enhancement in the North China’s Sea, Ministry of Agriculture and Rural Affairs at Dalian Ocean University (38°81′ N, 121°39′ E). To adapt to the laboratory environment, sea cucumbers were randomly distributed among four stocked tanks (80 cm × 50 cm × 55 cm) and were fed daily for five days. During the acclimation period, all tanks were supplied with filtered seawater under constant aeration, and received adequate food. The food consisted of sea mud mixed with a commercial diet in a 4:1 ratio. The commercial diet was provided by Anyuan Industrial Co., Ltd. (Dalian, China). Any uneaten food and feces were cleared from the tanks each day. Additionally, 25% of the seawater volume in every tank was replaced daily. A portable water quality meter (YSI Incorporated, Yellow Springs, OH, USA) was used to measure water quality parameters daily for five days prior to the start of the experiment. Dissolved oxygen was 7.46 ± 0.08 mg/L, salinity 29.14 ± 0.04, pH 8.06 ± 0.02 and water temperature 8.46 ± 0.03 °C (mean ± SE).

### 2.2. Experiment Design

Three distinct types of stressors were applied to sea cucumbers to induce the specific stress response patterns. These stressors were based on our previous method [9], and consisted of exposure to conspecific carcasses, seawater rinsing, and mechanical disturbance. In *A. japonicus*, a coelomic cavity containing coelomic fluid lies between the body wall and the visceral mass [24]. This unique body structure enables neurotransmitters, neuropeptides, and hormones to be released into the coelomic fluid and distributed via it to act as systemic signals. During stress responses, coelomocytes continuously exchange molecular signals with the neuroendocrine system and the musculature. Each group contained three individuals, and coelomocytes from each individual were separately subjected to transcriptomic analysis.

**Figure 1:**
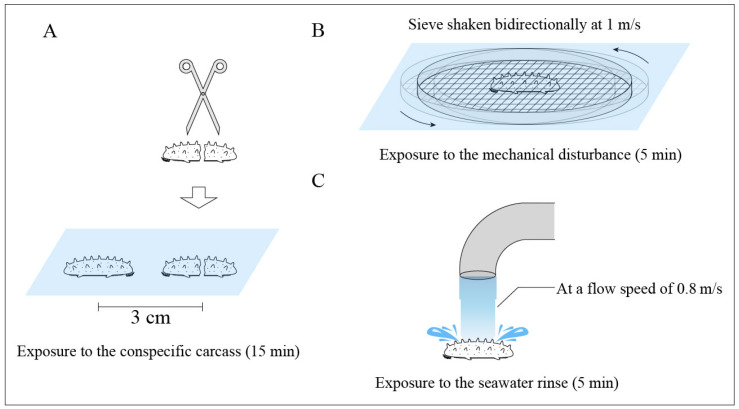
Sea cucumbers were exposed to conspecific carcasses (**A**), mechanical disturbance (**B**), and seawater rinsing (**C**), respectively.

Sea cucumbers were induced to exhibit the fleeing pattern by exposure to conspecific carcasses, following the experimental procedure below. One sea cucumber perished from oxygen deprivation in a plastic box without aeration (length × width × height: 20 cm × 20 cm × 15 cm). Its carcass was then bisected and used as a stressor of conspecific carcass. The conspecific carcass was positioned on the floor of the container (length × width × height: 45 cm × 33 cm × 10 cm). Meanwhile, the experimental sea cucumber was placed at a distance of 3 cm from the dead conspecific stressor, and this exposure lasted for 15 min (Figure 1A). The sea cucumbers induced into the fleeing pattern by using the above method were designated as group F. Sea cucumbers were induced to exhibit the adhesion pattern by exposure to a 5 min rinse of seawater at a flow speed of 0.8 m/s (Figure 1C). The sea cucumbers induced into the adhesion pattern by using the above method were designated as group A. Sea cucumbers were induced to exhibit the thanatosis pattern by mechanical disturbance following experimental procedure below. The experimental procedure involved putting a sea cucumber into a plastic sieve and then shaking the sieve bidirectionally at a speed of 1 m/s in seawater for 5 min (Figure 1B). Sea cucumbers that entered the thanatosis pattern via the above procedure were assigned to group T. Individuals not subjected to any stressor served as the control group (group C).

### 2.3. Sample Collection

These coelomocytes samples were collected immediately upon stressor removal. Sea cucumbers from each group were dissected along their ventral sides on an ice surface. Coelomic fluid was collected into 1.5 mL centrifuge tubes using sterilized syringes. Subsequently, the collected samples were centrifuged at 3000 rpm for 10 min using a refrigerated centrifuge (Eppendorf AG, Hamburg, Germany) at 4 °C. The coelomocytes at the bottom of the centrifuge tubes were retained and kept in a −80 °C freezer for subsequent analysis.

### 2.4. Transcriptomic Analyses

#### 2.4.1. RNA Extraction, Library Construction and Transcriptome Analyses

Following the instructions provided by the manufacturer, the total RNA was isolated from coelomocytes with TRIzol reagent (Invitrogen, Carlsbad, CA, USA). The concentration and purity of the RNA were assessed on an Agilent 2100 Bioanalyzer system (Agilent Technologies, Santa Clara, CA, USA). The RNA integrity number (RIN) of all samples exceeded 7.0. Following quality control validation, mRNA enrichment was carried out with oligo(dT)-conjugated magnetic beads. The enriched mRNA was then fragmented into pieces of appropriate size using a fragmentation buffer. First-strand cDNA was synthesized from the enriched mRNA using random hexamer primers through reverse transcription. Subsequently, the second-strand cDNA was synthesized to form double-stranded DNA. Next, the DNA fragments were subjected to end repair, A-tailing (adenylation), adaptor ligation, and PCR amplification. Finally, the PCR products were purified to obtain the final library. Following circularization, the single-stranded library was subjected to rolling circle amplification (RCA) using phi29 polymerase to generate DNA nanoballs (DNBs), each containing over 300 copies of the original circularized molecule. These DNBs were then deposited onto a patterned nanoarray, and paired-end 150 bp (PE150) sequencing was performed on the DNBSEQ platform using the DNBSEQ-T7 instrument (BGI-Shenzhen, China) in Wuhan.

#### 2.4.2. Read Mapping to the Reference Genome

The raw data were filtered using SOAPnuke (version 1.6.5) [25]. Clean reads were obtained after the removal of reads containing adapter sequences, reads containing poly-N, and low-quality reads from raw data. The filtered clean reads were saved in FASTQ format. Additionally, the Q20 and Q30 scores of the clean reads were calculated to assess sequencing quality. These high-quality clean reads served as the basis for all subsequent analyses. The reference genome and annotation files of the sea cucumber (*A. japonicus*) were obtained from the website at https://doi.org/10.1038/s41597-023-02368-9 [26]. HISAT (version 2.2.1) was employed to map the clean reads to the reference genome [27]. To identify novel transcripts, the genome-aligned reads were assembled into transcripts using StringTie (version 2.2.1) for each sample. All assembled transcripts were merged with Cuffmerge and compared to the reference annotation with Cuffcompare. Those with class codes “u”, “i”, “o”, or “j” were defined as novel. A custom reference transcriptome was then constructed by combining the known reference transcripts and the novel transcripts. Subsequently, the quality-controlled clean data were aligned to the custom reference transcriptome (including known and novel transcripts) using Bowtie2 (version 2.4.5) [28], and the gene expression levels in each sample were calculated using the RSEM (version 1.3.1) software package [29].

#### 2.4.3. Differential Gene Expression Analysis

DESeq2 (version 1.40.2) was employed to compare gene expression levels across different groups [30], with significance thresholds set at a |log_2_ fold change| ≥ 1 and a Q value (adjusted *p*-value) ˂ 0.05. A heatmap of the DEGs was generated using the heatmap function in software R (version 4.4.2) to visualize clustering patterns.

#### 2.4.4. GO and KEGG Enrichment Analysis of DEGs

Functional categorization of the DEGs was conducted based on Gene Ontology (GO) and Kyoto Encyclopedia of Genes and Genomes (KEGG). Enrichment analyses for KEGG pathways and GO terms were performed using the phyper function in software R (version 4.4.2). Pathways with a Q-value ˂ 0.05 were deemed to exhibit statistically significant enrichment.

#### 2.4.5. Reverse Transcription-Quantitative PCR (RT-qPCR) Validation

To validate the RNA-seq results, we randomly selected 6 DEGs for RT-qPCR validation. Coelomocyte RNA was reverse-transcribed into cDNA using the FastKing One-Step gDNA Removal and cDNA First-Strand Synthesis PreMix KR118-02 (TIANGEN Biotech Co., Ltd., Beijing, China) according to the manufacturer’s instructions. Primers were designed using NCBI Primer-BLAST (https://www.ncbi.nlm.nih.gov/tools/primer-blast/, accessed on 25 April 2026), and all primer sequences were provided in Table 1. Real-time PCR was performed on a QuantStudio 1 Plus Real-Time PCR System (Thermo Fisher Scientific, Waltham, MA, USA) using the TIANGEN FP217-02 kit (TIANGEN Biotech Co., Ltd., Beijing, China). The thermal cycling conditions were as follows: initial denaturation at 95 °C for 2 min, followed by 40 cycles of denaturation at 95 °C for 5 s and annealing at 60 °C for 15 s. The *Cytb* gene was used as the reference gene for normalization of target gene expression [31]. The relative expression level of the target gene was calculated using the 2^−ΔΔCt^ method, and the results are presented as log_2_ fold change. The software SPSS (version 20) was used for data analysis. The Shapiro-Wilk test was used to assess normality. As the data were normally distributed, an independent samples *t*-test was used to compare the differences between groups. Statistical significance was set at *p* < 0.05.

## 3. Results

### 3.1. Transcriptome Sequencing and Assembly

Transcriptome sequencing was performed on coelomocyte samples from sea cucumbers in groups C, F, A and T, respectively. After quality control, we obtained approximately 79.55 Gb of sequencing data. Each sample produced an average of 6.63 Gb data, with Q20 exceeding 98.46%, Q30 above 95.06%, and a clean reads ratio greater than 97.92% (The data are presented in the Appendix A Table A1). Sequence mapping analysis revealed that the total mapping rate for all samples was between 84.21% and 87.19%, while the proportion uniquely mapped to the reference genome ranged from 76.52% to 81.27% (The data are presented in the Appendix A Table A2). These results indicate high quality of the sequencing and library construction, confirming that the samples are appropriate for further analysis.

### 3.2. Analysis of Differentially Expressed Genes (DEGs)

**Figure 2:**
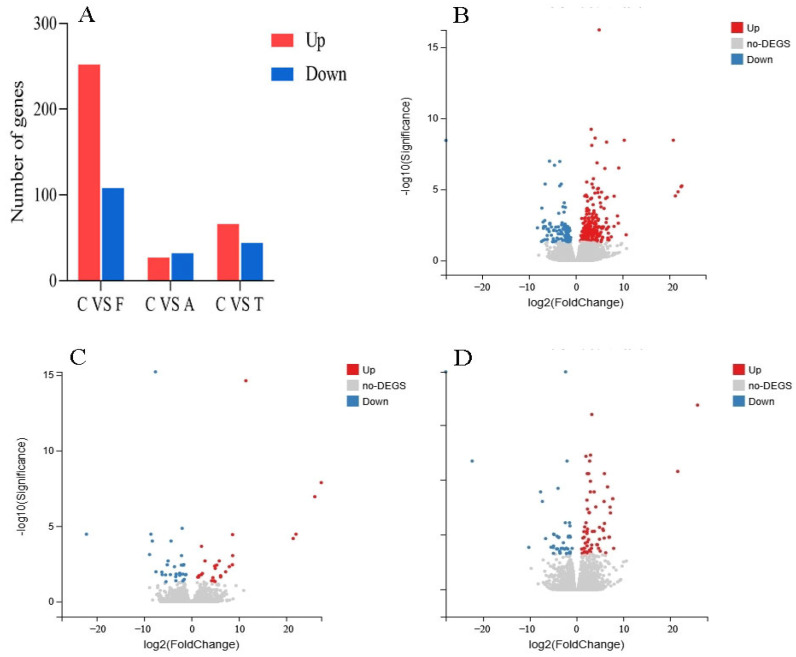
The number of differentially expressed genes (**A**), and the numbers of significantly up-regulated and down-regulated genes in groups F (**B**), A (**C**), and T (**D**).

**Figure 3:**
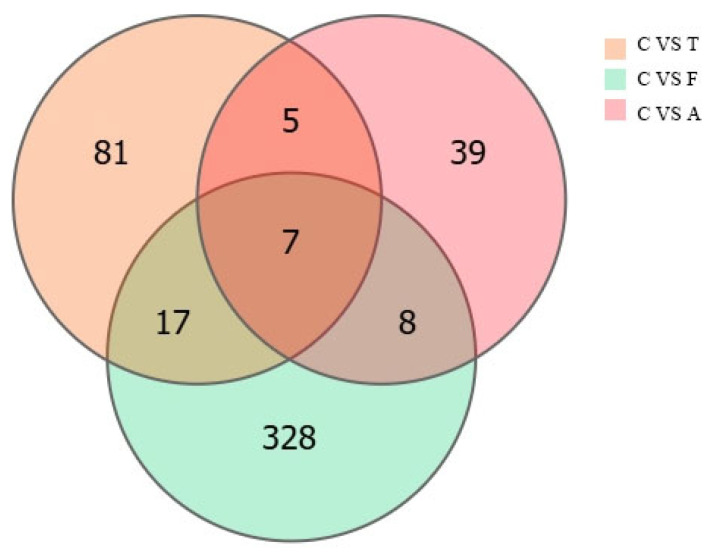
Venn diagram of differentially expressed genes among different comparison groups. C, T, F, A refer to groups C, T, F, A, respectively.

**Figure 4:**
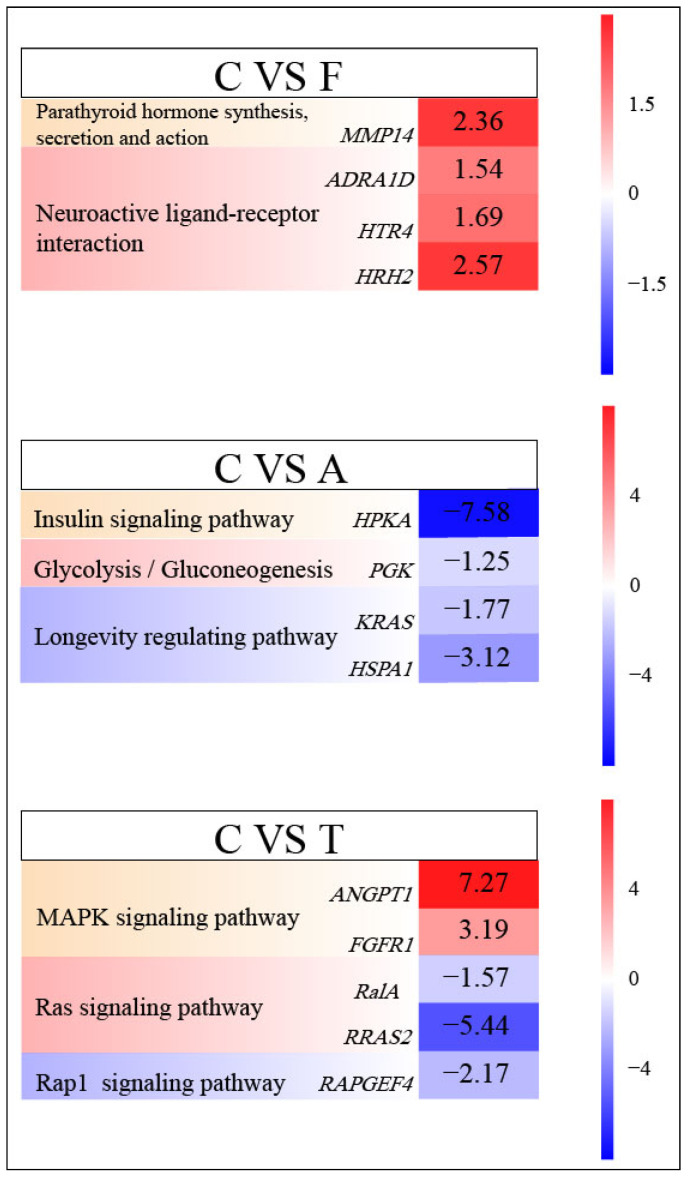
The names and expression trends (log_2_ fold change) of differentially expressed genes in the experimental group. C, T, F, A refer to groups C, T, F, A, respectively.

A total of 360 DEGs were identified in the comparison between group F and group C (Figure 2A). Among these, 252 genes were upregulated and 108 genes were downregulated (Figure 2B). The detailed results of the DEGs and their transcripts between groups F and C were provided in the Supplementary Materials S1 and S2, respectively. A comparison between groups A and C identified 59 DEGs (Figure 2A), comprising 27 upregulated and 32 downregulated genes (Figure 2C). The detailed results of the DEGs and their transcripts between groups A and C were provided in the Supplementary Materials S3 and S4, respectively. A total of 110 DEGs were identified in the comparison between groups T and C (Figure 2A). Among these, 66 genes were upregulated and 44 genes were downregulated (Figure 2D). Venn diagram analysis revealed that among these DEGs, 7 DEGs were shared by all three stress groups. The detailed results of the DEGs and their transcripts between groups T and C were provided in the Supplementary Materials S5 and S6, respectively. Regarding the overlaps between any two groups, 17 DEGs were shared between groups F and T. 8 DEGs were shared between groups F and A. 5 DEGs were shared between groups A and T. In addition, 328, 39, and 81 DEGs were uniquely identified in the groups F, A and T, respectively (Figure 3). This distribution pattern suggests that the three stress patterns trigger substantially distinct transcriptional reprogramming. Based on pathway analysis, genes significantly associated with the pathways were identified, and their expression levels (upregulated or downregulated) are presented in Figure 4.

### 3.3. GO Enrichment Analysis of DEGs

**Figure 5:**
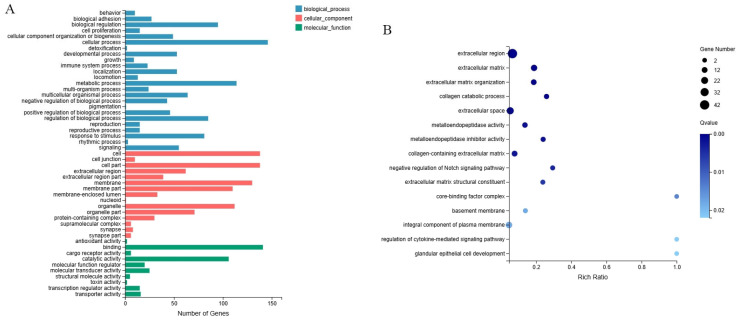
GO classification (**A**) and enrichment analysis (**B**) of differentially expressed genes between groups F and C.

GO enrichment analysis revealed that the DEGs in group F were primarily annotated to the three main GO categories: biological process, cellular component, and molecular function. Among biological process terms, cellular process predominated; the major cellular component terms were cell and cell part; and the major molecular function term was binding (Figure 5A). GO enrichment analysis identified 43 significantly enriched terms between groups F and C, comprising 32 biological processes, 7 cellular components, and 4 molecular functions. The top 15 enriched terms with the smallest Q-values are presented in Figure 5B.

**Figure 6:**
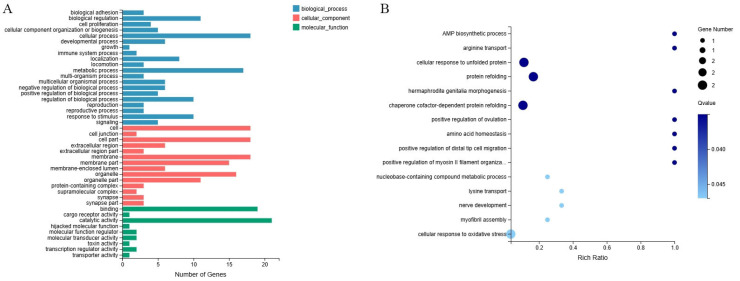
GO classification (**A**) and enrichment analysis (**B**) of differentially expressed genes between groups A and C.

GO enrichment analysis revealed that the DEGs in group A were primarily annotated to the three main GO categories: biological process, cellular component, and molecular function. Among the biological process terms, cellular process and metabolic process predominated; the major cellular component terms were cell, cell part, and membrane; and the most prominent molecular function terms were binding and catalytic activity (Figure 6A). GO enrichment analysis identified 42 significantly enriched terms between groups A and C, comprising 36 biological processes and 6 molecular functions. The top 15 terms with the smallest Q-values are presented in Figure 6B.

**Figure 7:**
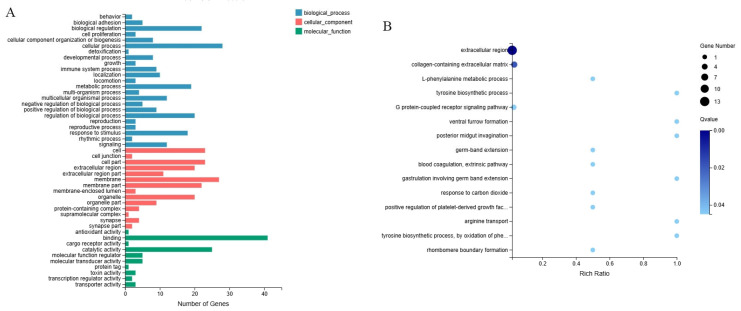
GO classification (**A**) and enrichment analysis (**B**) of differentially expressed genes between groups T and C.

GO enrichment analysis revealed that the DEGs in group T were primarily annotated to the three main GO categories: biological process, cellular component, and molecular function. Among biological process terms, cellular process and biological regulation were predominant. The major cellular component terms were membrane, cell, and cell part. The most prominent molecular function terms were binding and catalytic activity (Figure 7A). GO enrichment analysis identified 57 significantly enriched terms between groups T and C, comprising 55 biological processes and 2 cellular components. The top 15 terms with the smallest Q-values are presented in Figure 7B.

### 3.4. KEGG Enrichment Analysis of DEGs

**Figure 8:**
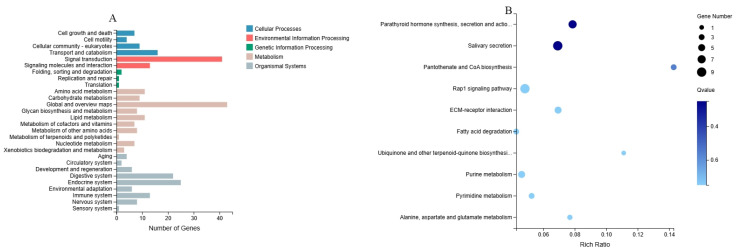
KEGG pathway classification (**A**) and enrichment (**B**) of differentially expressed genes between groups F and C.

KEGG analysis revealed that the DEGs in group F were primarily annotated in two functional categories: signal transduction under environmental information processing, and global and overview maps under metabolism (Figure 8A). The top 10 most highly enriched pathways between groups F and C are shown in Figure 8B. While several pathways showed nominal significance (*p* < 0.05), these did not remain significant after the FDR correction.

**Figure 9:**
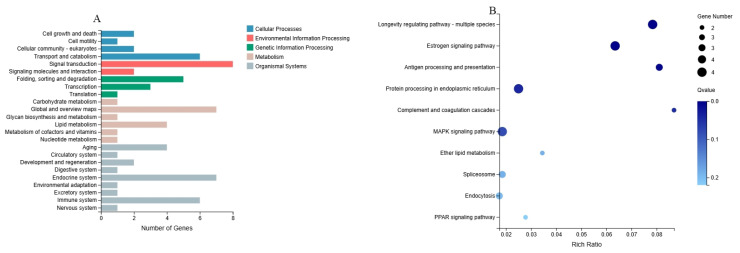
KEGG pathway classification (**A**) and enrichment (**B**) of differentially expressed genes between groups A and C.

KEGG analysis revealed that the DEGs in group A were primarily annotated three functional categories: signal transduction under environmental information processing, global and overview maps under metabolism and endocrine system under organismal systems (Figure 9A). The top 10 most highly enriched pathways between groups A and C were shown in Figure 9B. Among these, DEGs were significantly enriched in pathways such as the longevity regulating pathway, estrogen signaling pathway, antigen processing and presentation, protein processing in endoplasmic reticulum, and complement and coagulation cascades.

**Figure 10:**
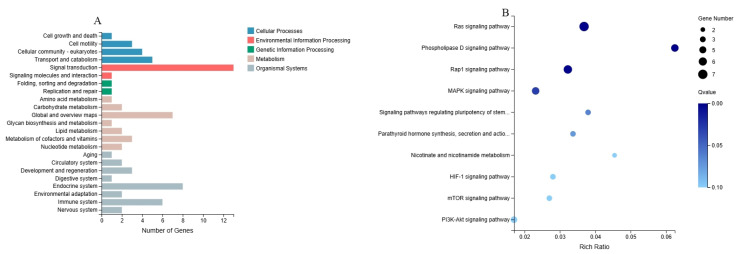
KEGG pathway classification (**A**) and enrichment (**B**) of differentially expressed genes between groups T and C.

KEGG analysis revealed that the DEGs in group T were primarily annotated three functional categories: signal transduction under environmental information processing, global and overview maps under metabolism and endocrine system under organismal systems (Figure 10A). The top 10 most highly enriched pathways between groups T and C were shown in Figure 10B. Among these, DEGs were significantly enriched in several pathways, including Phospholipase D, Ras, Rap1 and MAPK signaling pathways.

### 3.5. RT-qPCR Validation

**Figure 11:**
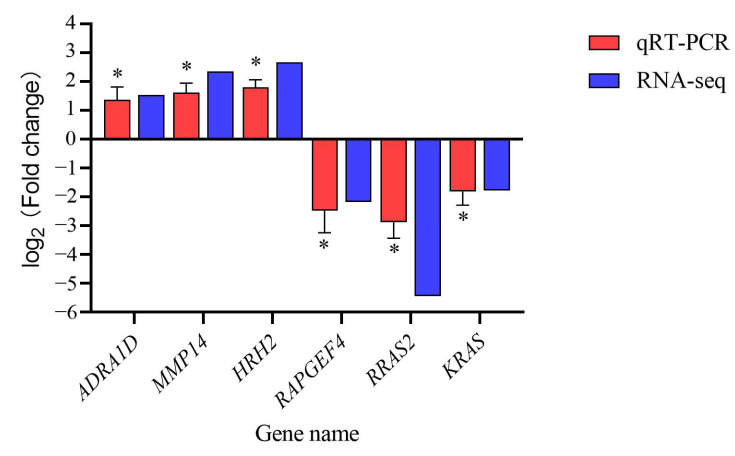
qRT-PCR validation of RNA-seq results for six differentially expressed genes (The asterisk * mean *p* ˂ 0.05).

The results of RT-qPCR and RNA-seq analyses for six genes were presented in Figure 11 (mean ± SE). The RT-qPCR confirmed that all six selected genes (*ADRA1D*, *MMP14*, *HRH2*, *RAPGEF4*, *RRAS2*, and *KRAS*) were significantly differentially expressed (*p* < 0.05), with expression trends consistent with the RNA-seq data. Thus, these RT-qPCR results confirmed the reliability of the RNA-seq data.

## 4. Discussion

### 4.1. Regulatory Mechanisms Potentially Driving the Fleeing Pattern

Fleeing is an important behavioral strategy employed by organisms to evade threats [32]. Organisms exhibit rapid and vigorous movement during the escape response [33]. Sea cucumbers exhibit a significantly increased crawling frequency and move faster when displaying the fleeing pattern [9]. Calcium ions are essential for sustained muscle contraction in high-intensity exercise [34]. In the present study, we found a consistent significant upregulation of several matrix metalloproteinase genes, including *MMP14*, *MMP15* and *MMP24*. The *MMP14* is transcriptionally regulated by parathyroid hormone signaling in osteocytes, and its protein product promotes bone resorption, a key process for releasing calcium from the bone into the bloodstream [35]. *Apostichopus japonicus* possesses an endoskeleton made of ossicles [24], which holds the potential to release calcium ions. These results suggest that the matrix metalloproteinase family, especially *MMP14*, may participate in the regulation of the fleeing pattern, possibly by modulating the availability of calcium ion to support continuous rapid movement.

Escape response is regulated by the concerted action of multiple neurotransmitters [10]. The present study found the significant upregulation of three genes *HTR4*, *ADRA1D,* and *HRH2*, which encode receptors for 5-HT, adrenaline/norepinephrine, and histamine, respectively. These results suggest that the fleeing pattern is not regulated by a single neurotransmitter alone, but rather involves a coordinated modulation of these multiple receptor systems in sea cucumbers. 5-HT receptors mediate the signal transduction of 5-HT [36]. They mediate and sustain the vigilance state induced by conspecific alarm substance in zebrafish *Danio rerio*, thereby increasing their sensitivity to threatening stimuli [37]. Consistently, sea cucumbers exhibited a flight reaction when being exposed to conspecific corpses, accompanied by significantly higher 5-HT levels [11]. These findings suggest that the activation of the 5-HT receptors may facilitate the perception of danger signals in sea cucumbers. The adrenergic receptors receive the signal from norepinephrine and epinephrine [38]. These catecholamines are involved in the physiological activation of the sympathetic nervous system [39], which is responsible for initiating the “fight or flight” response [40]. Furthermore, a significantly higher level of norepinephrine was observed in sea cucumbers exposed to conspecific corpses in fleeing pattern [9]. These results suggest that the adrenergic receptor plays a potentially important role in initiating the fleeing pattern. Histamine is an important neurotransmitter, and its effects are mediated through histamine receptors [41]. Histaminergic neurons specifically mediate accelerated motor behaviors during alertness in mice [42]. Histaminergic neurons are present in the buccal tentacles, circumoral nerve ring, and body wall papillae of the sea cucumber *Leptosynapta clarki*, and histamine significantly promotes the crawling frequency in them [43]. These findings suggest histaminergic neurons may participate in the crawling behavior in fleeing patten. In summary, the upregulation of *HTR4*, *HRH2*, and *ADRA1D* may play important roles in regulation of the fleeing pattern in sea cucumbers.

### 4.2. Potential Regulatory Mechanisms of Adhesion Pattern

Glycolysis is a crucial pathway for energy production [44]. Under stress conditions, glycolytic flux is modulated to reorganize cellular energy metabolism [45,46]. In the present study, the expression of *PHKA* and *PGK* was significantly downregulated in adhesion pattern (group A). Both genes play important roles in the glycolysis. Phosphorylase kinase plays a critical role in initiating glycogenolysis by activating glycogen phosphorylase via phosphorylation [47,48]. Phosphoglycerate kinase participates in the first ATP-generating step of glycolysis [49,50]. These results suggest that there was a reduced glycolytic flux in the adhesion pattern. According to existing studies, such physiological changes suggest an energy-conserving strategy that prioritizes the allocation of limited energy to vital tissues [51,52]. Consistent with this strategy, sea cucumbers may allocate a greater proportion of available energy to vital tissues to sustain the adhesion pattern. In addition, the present transcriptomic analysis revealed that the DEGs were significantly enriched in the longevity regulating pathway in adhesion pattern (group A), with a significant downregulation of *KRAS* and *HSPA1*. Cell proliferation and differentiation require a continuous energy supply and are temporarily arrested when energy reserves become limited [53,54]. *KRAS* serves as a key regulator of cell proliferation and differentiation [55,56], and inhibition of the signaling leads cells to rapidly cease proliferation and enter a reversible and quiescent state [57]. These findings suggest that cell proliferation and differentiation may be reduced as part of an adaptive strategy to reallocate limited energy resources. Heat shock protein (HSP) family genes are frequently transcriptionally upregulated to enable organisms to withstand stressful environments [58,59]. However, the synthesis and functional maintenance of HSPs represent an energetically costly process [60,61]. Therefore, organisms downregulate the expression levels of certain HSP family members to optimize energy allocation under energy-limited conditions. For example, *HSP70* expression was significantly reduced in brown adipose tissue of hibernating thirteen-lined ground squirrel *Spermophilus tridecemlineatus* [62]. Similarly, a significant downregulation of *HSP70* mRNA was observed in the liver of hibernating chipmunk *Tamias asiaticus* [63]. The significant downregulation of *HSPA1* may be an energy allocation strategy to adapt the limited energy supply. In summary, sea cucumbers appear to reallocate energy resources by suspending energy-intensive biological processes to sustain adhesion pattern.

### 4.3. Regulatory Mechanisms Underlying Thanatosis Pattern

Body wall musculature of sea cucumbers mainly consists of an outer longitudinal and an inner circular smooth muscle layer [64]. Sea cucumbers curl up their bodies in the thanatosis pattern [9]. The maintenance of this contracted posture requires the coordinated involvement of both circular and longitudinal smooth muscles. In the present study, the transcriptomic analysis revealed that the DEGs were significantly enriched in the MAPK signaling pathways in thanatosis pattern (group T), with significant upregulation of *ANGPT1* and *FGFR1*. Meanwhile, the expression of *RhoA* was significantly downregulated in group T. RhoA is a key regulator of smooth muscle contraction, and its activation is necessary for various smooth muscle contractions [65]. This seemingly contradictory combination of signals may result from the diversity of regulatory mechanisms governing smooth muscle contraction. The RhoA/ROCK pathway drives contraction by inhibiting myosin light chain phosphatase (MLCP) to enhance calcium sensitization [66]. However, the MAPK pathway can participate in the regulation of contraction independently of RhoA. Dessy et al. (1998) demonstrated that MAPK activation directly induces Ca^2+^-independent smooth muscle contraction [67]. The underlying mechanism involves MAPK (particularly ERK1/2) phosphorylating the actin-binding protein caldesmon, causing its dissociation from F-actin. This relieves caldesmon-mediated inhibition of actin–myosin interaction, thereby permitting cross-bridge formation and tension maintenance [68]. *ANGPT1* and *FGFR1* both potently activate the MAPK signaling pathway [69,70]. These results suggest that the MAPK pathway and *RhoA* may be jointly involved in sustaining body wall smooth muscle contraction during the thanatosis pattern in sea cucumbers.

Organisms reallocate energy to cope with various stress states [71]. Glucose is the primary source of energy [72]. Acute stress may disrupt glucose homeostasis, leading to dramatic fluctuations in blood glucose levels [73]. In the present study, transcriptomic analysis revealed that the DEGs were significantly enriched in the Rap1 signaling pathway in group T, with the significant downregulation of the *RAPGEF4*. *RAPGEF4* is a key activator of Rap1. Inhibition of central Rap1 activity significantly reduces blood glucose levels in mice [74]. Consistently, Wang et al. (2025) reported that sea cucumbers subjected to mechanical disturbance for 10 min exhibited significantly reduced glucose levels (the same method for inducing sea cucumbers into thanatosis pattern) [14]. The significant downregulation of the *RAPGEF4* in Rap1 signaling pathway suggests that *RAPGEF4* may be involved in decreased glucose levels under thanatosis pattern. In addition, the present transcriptomic analysis revealed that the DEGs were significantly enriched in the Ras signaling pathway in group T, with the significant downregulation of the genes of *RRAS2* and *RalA*. *RRAS2* directly binds to and activates PI3Kα, accelerating cellular glucose uptake [75]. *RalA* regulates glucose homeostasis by promoting glucose uptake in brown adipose tissue [76]. The downregulation of these genes indicates a reduction in the efficiency of cellular glucose uptake. This may be an adaptive adjustment to the decreased glucose availability. In addition, sea cucumbers displayed a tendency to remain stationary after the termination of the thanatosis pattern [9]. The stationary tendency may be attributed to the failure to restoring glucose homeostasis.

### 4.4. Limitations of the Study

These stress response patterns were induced by the specific stressor type, intensity, and duration used in this study. Whether they would change under different stress conditions remains unclear, and this will be investigated in future study. In addition, it is valuable to conduct transcriptomic profiling of neural and muscle tissues from sea cucumbers in different stress response patterns, with the aim of gaining deeper insights.

## 5. Conclusions

Fleeing, adhesion, and thanatosis are common stress response patterns to various types of stressors in sea cucumbers. RNA-seq results revealed that the fleeing pattern is potentially regulated by the *HTR4*, *HRH2*, *ADRA1D* and *MMP14*. In the adhesion pattern, sea cucumbers may reallocate energy resources to maintain adhesion by suppressing energy-intensive processes via downregulation of the longevity-regulating pathway and *PGK*. In the thanatosis pattern, MAPK pathway potentially regulates muscle contraction to maintain the contracted posture. The Rap1 and Ras pathways potentially participate in the reduction in glucose and the adaptations, respectively. The present study offers valuable insights into comprehending the potential molecular regulatory mechanisms of the fleeing, adhesion, and thanatosis patterns in sea cucumbers.

## Figures and Tables

**Table 1:** Sequences of primers used for quantitative real-time PCR.

Gene Name	Primer Sequence (5′-3′)
*ADRA1* *D*	F: TACGTCCTGTGCGCTTTCAT
	R: AGGAGCAAAAGGTGCGAGAA
*MMP14*	F: GGAGCGGTAAAGGTCCACAA
	R: GCCGATGGACACCTCAGAAA
*HRH2*	F: TGGTGGCGTCTCAACTCATC
	R: CCATCATTCTCCTGCCGTGT
*RAPGEF4*	F: CGGAGAGCGTTGGCGATTCT
	R: GGAACTCCAGCCATCACAGAG
*RRAS2*	F: GCATTGGAGTTGGAAGGGGA
	R: CCGTTCTTGGCCTACCTTGT
*KRAS*	F: CCTGGACGGTGGATAGCAAA
	R: TTGCTACTGGCGTTTCGTCT
*Cytb*	F: TGAGCCGCAACAGTAATC
	R: AAGGGAAAAGGAAGTGAAAG

## Data Availability

The transcriptomic data have been deposited with links to BioProject accession number PRJNA1467255 in the NCBI BioProject database (https://www.ncbi.nlm.nih.gov/bioproject/, accessed on 18 May 2026).

## Supplementary Materials

The following supporting information can be downloaded at: https://www.mdpi.com/article/10.3390/biology15120975/s1, The detailed results of the DEGs and their transcripts between groups F and C were provided in the Supplementary Materials S1 and S2, respectively. The detailed results of the DEGs and their transcripts between groups A and C were provided in the Supplementary Materials S3 and S4, respectively. The detailed results of the DEGs and their transcripts between groups T and C were provided in the Supplementary Materials S5 and S6, respectively.

## Appendix A

**Table A1:** Quality control and data statistics for clean reads.

Sample	Raw Reads (M)	Clean Reads (M)	Clean Bases (Gb)	Q20 (%)	Q30 (%)	Clean Reads Ratio (%)
C01	22.56	22.15	6.64	98.46	95.06	98.18
C02	22.56	22.11	6.63	98.59	95.5	98.01
C03	22.56	22.11	6.63	98.65	95.66	98.01
F01	22.4	22.02	6.61	98.61	95.54	98.3
F02	22.56	22.11	6.63	98.59	95.47	98.01
F03	22.4	22.03	6.61	98.59	95.46	98.35
A01	22.56	22.13	6.64	98.55	95.33	98.09
A02	22.56	22.14	6.64	98.68	95.74	98.14
A03	22.56	22.14	6.64	98.5	95.15	98.14
T01	22.56	22.11	6.63	98.62	95.53	98.01
T02	22.4	22.06	6.62	98.72	95.9	98.48
T03	22.56	22.09	6.63	98.53	95.25	97.92

**Table A2:** Sequence mapping analysis.

Sample	Total Clean Reads (M)	Total Mapping (%)	Uniquely Mapping (%)
C01	22.15	86.76	81.27
C02	22.11	86.76	80.23
C03	22.11	84.21	78.88
F01	22.02	86.78	80.9
F02	22.11	86.55	80.29
F03	22.03	86.84	78.16
A01	22.13	87.19	80.9
A02	22.14	85.21	78.75
A03	22.14	86.83	81.26
T01	22.11	85.48	76.52
T02	22.06	86.64	79.88
T03	22.09	86.69	80.16
